# B memory cell responses to LPS, IVP and IpaB antigen after oral vaccination with *Shigella sonnei* vaccine candidates WRSs2 and WRSs3

**DOI:** 10.1371/journal.pone.0290987

**Published:** 2024-01-17

**Authors:** Malabi M. Venkatesan, Shoshana Barnoy, Robert Frenck, Monica McNeal, Shahida Baqar

**Affiliations:** 1 US Army Bacterial Diseases Branch, Walter Reed Army Institute of Research, Silver Spring, Maryland, United States of America; 2 Division of Infectious Diseases, Department of Pediatrics, Cincinnati Children’s Hospital Medical Center, University of Cincinnati College of Medicine, Cincinnati, Ohio, United States of America; 3 Division of Microbiology and Infectious Diseases, National Institute of Allergy and Infectious Diseases, National Institutes of Health, Bethesda, Maryland, United States of America; Universidad Nacional de la Plata, ARGENTINA

## Abstract

B memory (B_M_) cell responses were evaluated using peripheral blood mononuclear cells that were collected and cryopreserved during a Phase 1 trial of two live *Shigella sonnei* vaccine candidates WRSs2 and WRSs3. An ELISpot assay was used to measure IgG+ and IgA+ B_M_ cell responses against *S*. *sonnei* LPS, IVP and IpaB antigens. Analysis of B_M_ cell responses at baseline, and on days 28 and 56 post vaccination indicate that after a single oral dose of WRSs2 and WRSs3, both groups of vaccinees induced IgG+ and IgA+ B_M_ cell responses that were variable in magnitude among subjects and reached significance to IVP and IpaB at several doses. The responses generally peaked at d28 after vaccination. The baseline as well as post-vaccination levels of IgA+ B_M_ cells were relatively higher than IgG+ B_M_ cells, but the maximum fold-increase at d28/d56 over baseline was greater for IgG+ than IgA+ B_M_ cell responses. Furthermore, at the three highest vaccine doses, >60–90% of subjects were considered responders indicating a ≥2-fold higher IgG+ B_M_ cell responses to IVP and IpaB post vaccination, while fewer subjects indicated the same level of response to LPS.

## Introduction

*Shigella* is an enteroinvasive bacterial pathogen that causes diarrhea and dysentery, and shigellosis constitutes an important cause of morbidity and mortality in children less than five years of age living in low income countries. Multiple serotypes of *Shigella* can cause disease and in the face of rising antibiotic resistance, both live attenuated as well as subunit vaccine candidates are undergoing testing in clinical trials. The primary antigen targeted for vaccine-induced protection is centered around the bacterial outer membrane-anchored lipopolysaccharide (LPS) and the surface-localized invasion plasmid antigens or Ipa antigens. Serum IgG antibodies to LPS as well as antibodies to IpaB have been implicated in protection against shigellosis [1].

Live, attenuated *Shigella sonnei* vaccine candidates WRSs2 and WRSs3 were recently evaluated in a Phase 1 placebo-controlled dose-finding study and shown to be safe and immunogenic (2, 3). The primary attenuating feature of these vaccine candidates is the inability to spread intercellularly due to lack of VirG(IcsA), thereby reducing the inflammatory potential of these strains. A previous version WRSS1, that lacked only VirG(IcsA), was safe at 10^4^ CFU but showed mild and transient diarrheal symptoms and fever at higher doses. In order to reduce the adverse symptoms seen with WRSS1, enterotoxin gene *senA* and its paralog *senB* were deleted from WRSs2 and WRSs3. Additionally WRSs3 lacks one of the two *msbB* genes that ensures maximal endotoxicity of *Shigella* LPS [2, 3]. WRSs2 and WRSs3 were administered as single, oral doses ranging sequentially from 10^3^−10^7^ CFU to 8 subjects /dose and a total of 9 subjects were placebos receiving saline [2, 3]. Immunogenicity data indicated that there were higher serum and mucosal IgA responses to LPS, IVP and IpaB compared to IgG responses. Mucosal immune responses were measured as antigen-specific antibody secreting cells (ASCs), antibody in lymphocyte supernatants (ALS) and fecal IgA (2, 3). WRSs2 vaccinees had higher magnitude of responses as well as responder rates than WRSs3 vaccinees, although associations between the different immune categories were similar [3]. Though both vaccine candidates were safe at the highest tested dose, the immunogenicity data suggested that WRSs2 would be advanced for further clinical studies (NCT04242264).

While serum and mucosal antibodies to *S*. *sonnei* LPS, IVP and IpaB are important elements contributing to the hosts immune defense against this pathogen, persistence of these immune responses after a single dose was not evident by assays employed [2, 3]. The antigen-driven conversion of naïve B cells to long lived plasma cells occurs in B cell follicles and germinal centers and generates protective antibodies and B memory (B_M_) cells that respond to reinfection [4, 5].The molecular mechanisms leading to the development and maturation of B_M_ cells is currently an intensive area of investigation. Long-term protection against shigellosis will likely require the induction of B_M_ and T cells during an initial infection that are capable of an accelerated, robust anamnestic immune response during reinfection. Here we describe the induction of IgG+ and IgA+ B_M_ cell responses to *S*. *sonnei* LPS, IVP and IpaB after a single dose of WRSs2 and WRSs3 using an ELISpot assay (cell-based enzyme-linked immunospot assay).

## Materials and methods

### Study samples and antigens

Safety and immunogenicity of WRSs2 and WRSs3 in a Phase 1 clinical trial has been previously described and includes sample collection and assay procedures for measuring serum and mucosal responses [2, 3]. B_M_ cell responses were evaluated using available cryopreserved PBMCs with a minor modification of methods previously published [6, 7]. LPS, Invaplex50 (IVP) and purified IpaB protein were obtained from Dr. Robert Kaminski at WRAIR. LPS was purified from *S*. *sonnei* using the hot aqueous phenol extraction method. *S*. *sonnei* Invaplex50 (IVP) is composed mainly of *S*. *sonnei* LPS, IpaB and IpaC, although other proteins are also present. Purified IpaB was obtained by affinity chromatography over nickel columns using histidine-tagged IpaB protein.

### ELISpot assay for measuring B_M_ cell responses

B_M_ cell responses were evaluated using minor modification of methods previously published [6, 7, 8]. The antigen-specific (LPS, IVP, IpaB) IgG+ and IgA+ B_M_ cell responses and total IgA+ and IgG+ B_M_ cell responses using expanded cells were measured by an ELISpot assay briefly described below [8].

After thawing and recovering PBMCs overnight at 37° in complete media with 5% CO_2_, live cells were adjusted to 4x10^6^ PBMCs per mL in CTL medium (CTL # TB-005) containing 1% L-glutamine and B-Poly-S reagent (CTL # BPOPYS-200). 1 mL cell suspension were plated in 24-well tissue culture plate and incubated at 37°C, 5% CO_2_ for 4 days. Following incubation, cells were collected, washed and viability counted, and expansion was considered adequate if final viable cell concentration for each sample was within 20% of the starting concentration. Expanded memory cells were diluted to 2x10^6^ cells/ml and added to antigen-coated plates. Two-fold dilutions were made in duplicate for antigen-specific (2.5x10^5^ cells/well) and total IgA/IgG wells. Cells making antibodies were enumerated using isotype specific human biotinylated αIgG and αIgA antibodies and developed with Extravidin Peroxidase and AEC substrate. Plates were read in a CTL ELISpot reader and spot forming cells (SFCs) counted. Duplicate wells were averaged and expressed as SFCs/10^6^ expanded PBMCs. The B_M_ cell response at baseline (d-1), d28 and d56 for each subject was expressed as the percentage of antigen-specific IgG+ or IgA+ B_M_ cells out of the corresponding total IgA+ or IgG+ B_M_ SFCs. Those responses that showed a zero value were given a value of 0.001 which was taken as the minimum limit of detection. Subjects showing a ≥2-fold increase in antigen-specific B_M_ cells at d28 and d56 over baseline were considered responders in this study.

### Statistical analysis

A cohort B_M_ cell response was described by the geometric mean (GM) of the maximum fold increase (d28 or d56) compared to baseline levels of each subject in the cohort and standard deviation (SD) of the maximum fold increase was calculated to obtain GM ± SD. The kinetics of antigen-specific IgA+/IgG+ B_M_ cell response was derived by obtaining GM of the IgA+/IgG+ B_M_ cell responses at baseline and post vaccination days for each vaccine and dose group. The significance of the IgA+/IgG+ B_M_ cell responses induced at d28 and d56 was calculated using a paired *t*-test (*p* value of <0.05). The relationship between immunological parameters at the three highest vaccine doses was assessed using ANOVA linear regression analysis (Pearson coefficient) of the log10 transformed values of the maximum B_M_ cell responses which were correlated with the corresponding values for IgA/IgG serum antibodies, ASCs, ALS and fecal IgA as described previously [3].

### Ethical review

The clinical study described earlier was reviewed and approved by the CCHMC IRB (IRB #FWA00002988) and conducted according to the standards of ICH-GCP E6, under a US Food and Drug Administration-approved IND [2, 3]. The authors did not have access to information that could identify individual participants during or after data collection. The investigators have adhered to the policies for protection of human subjects as prescribed in AR 70–25. The authors in this manuscript have no conflicts of interest. MMV was and SBaqar is a U.S. government employee.

## Results

### Magnitude of B_M_ cell responses after a single oral dose of vaccination

**Fig 1:**
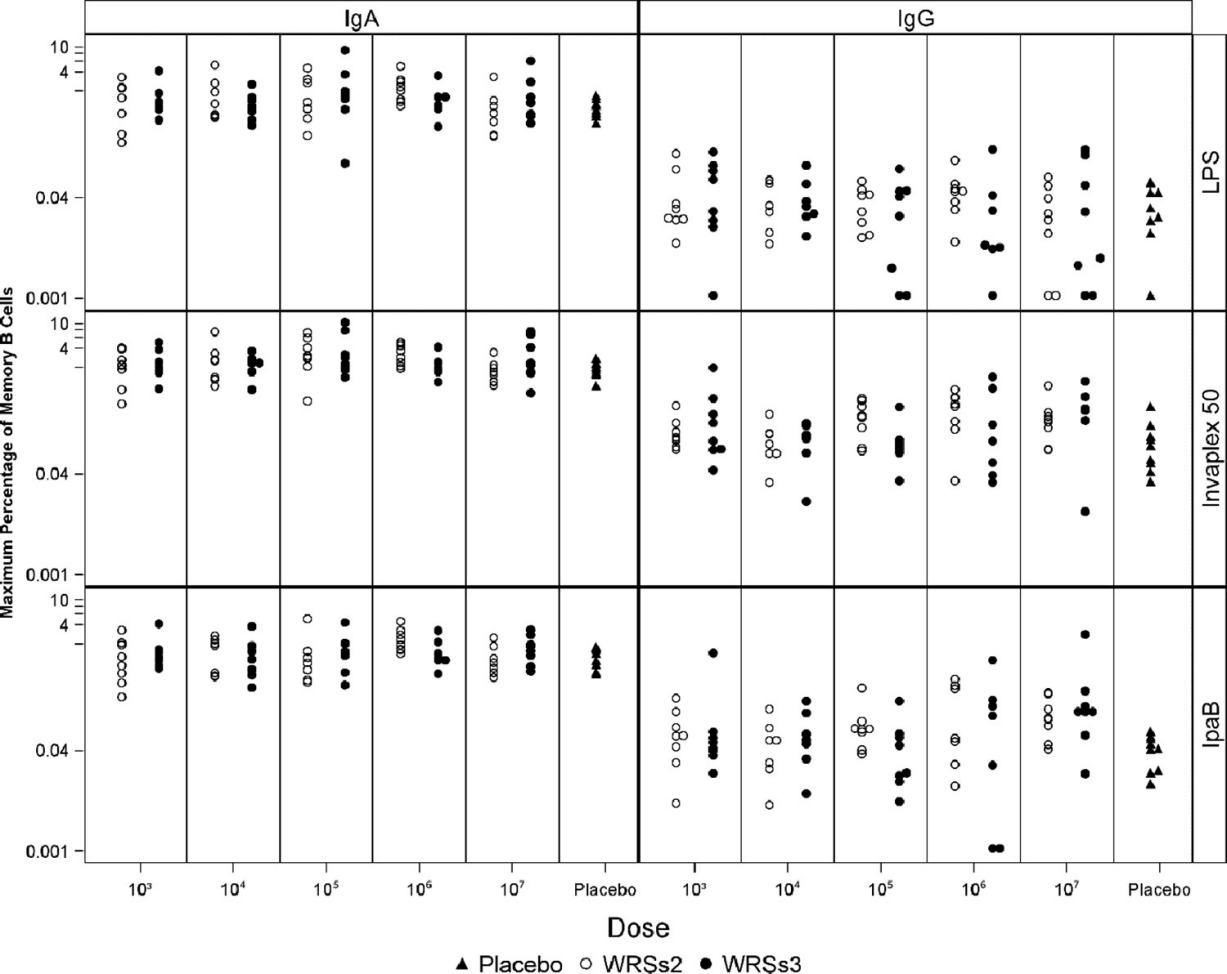
Maximum % of B_M_ cell responses after WRSs2 & WRSs3 vaccination. Maximum % of antigen-specific IgG and IgA+ B_M_ cell responses of each subject after WRSs2/ WRSs3/placebo administration is given on the Y axis and the X axis provides the CFU doses of the vaccine candidates from 10^3^ to 10^7^ CFU. The antigens used (LPS, IVP and IpaB) are mentioned on the right side of the figure and the isotype (IgA or IgG) of the B_M_ cell response is indicated at the top. For all doses in both groups of vaccinees, the number of archived samples available were n = 8, except the following; WRSs2 10^4^ CFU for all time points n = 7; WRSs3 10^4^ CFU n = 7, 7, 6 for d-1, d28, d56; WRSs3 10^5^ CFU n = 7 for d56; WRSs3 10^6^ CFU n = 6 for d-1, d28, d56; and WRSs3 10^7^ CFU n = 7 for d-1. For placebo group available samples were n = 9, 8 and 9 at 3 time points.

The maximal B_M_ cell response of each subject (at either d28 or d56) in both groups of vaccinees across all 5 doses is shown in Fig 1. The placebo responses reflect the variability in baseline levels of B_M_ cells and is higher for IgA+ than IgG+ B_M_ cells. In general, the magnitude of the IgA+ B_M_ cell responses at baseline, ranging from 0.3 to >1.0, was almost one to two orders of magnitude higher than the corresponding levels of baseline IgG+ B_M_ cell responses which was in the range of 0.002–0.08. Thus, post vaccination, the range in the magnitude of IgA+ B_M_ cell responses across all 5 doses and for all 3 antigens in both groups of vaccinees was higher than the corresponding IgG+ B_M_ cell responses (Fig 1). Importantly the vaccinee responses among subjects in both groups also varied in magnitude (Fig 1).

Although the maximal IgA+ B_M_ cell responses were higher than IgG (Fig 1), the maximum fold-increase (d28/d-1 and d56/d-1) of the IgG+ B_M_ cell responses was higher than the corresponding fold-increase of IgA+ B_M_ cell responses across all vaccine doses. The geometric mean (GM ± SD) of the maximum fold-increase at each dose for each antigen is shown in Table 1. Although a dose response is not clearly evident, all three antigens increased IgG+ B_M_ cell responses over baseline and the fold-increase was generally greater at the three highest doses and above what is seen with the placebos. Generally the highest and the most consistent IgG+ B_M_ response was to IVP and IpaB among both groups although at 10^6^ CFU the LPS-specific IgG+ B_M_ response was the highest among WRSs2 vaccinees. In fact the peak GM of the maximal fold-increase to LPS and IVP IgG+ B_M_ cell responses was seen at the 10^6^ CFU dose for both vaccine candidates (Table 1). Comparatively, the maximum fold-increase was much lower for the IgA+ B_M_ cell response and at the lower doses the fold-increase for the IgA+ B_M_ cell response to all 3 antigens was closer to the fold-increase seen in placebos (Table 1).

**Table 1:** Geometric mean of B_M_ cell responses after WRSs2 & WRSs3 vaccination.

		10^3^ CFU		10^4^ CFU		10^5^ CFU		10^6^ CFU		10^7^ CFU		Placebo
		WRSs2	WRSs3	WRSs2	WRSs3	WRSs2	WRSs3	WRSs2	WRSs3	WRSs2	WRSs3	Placebo
**IgG+**	**LPS**	5.41±3.41	2.73±4.18	3.26±2.36	2.66±11.49	1.53±2.26	3.13±3.14	13.50±6.51	3.95±2.96	6.47±9.32	1.51±3.23	1.28±5.89
		50	50	43	57	25	63	75	43	57	43	11
	**IVP**	2.93±4.23	1.44±1.71	2.36±1.47	1.82±2.39	4.42±2.01	4.58±4.16	7.53±2.29	6.76±3.19	7.43±5.09	3.10±2.04	1.07±4.69
		50	25	57	29	75	63	88	86	71	71	22
	**IpaB**	6.08±5.09	4.84±4.39	4.43±3.44	7.06±1.99	6.08±3.38	13.87±2.89	3.75±3.59	8.03±7.41	14.86±7.55	7.45±4.24	1.93±9.64
		63	50	71	100	88	88	63	57	71	86	22
**IgA+**	**LPS**	1.45±1.83	1.53±1.59	1.28±2.57	2.51±1.92	1.92±1.83	3.03±2.58	2.12±2.15	1.71±1.79	1.72±1.41	1.62±1.67	1.49±1.77
		13	25	14	57	63	50	63	29	29	29	33
	**IVP**	1.52±2.25	1.49±1.60	1.73±1.50	2.34±1.83	2.87±1.90	2.92±1.80	1.78±1.42	1.84±1.64	1.66±1.95	1.90±1.82	1.23±2.18
		25	38	14	57	75	50	38	43	38	43	22
	**IpaB**	1.44±2.10	0.94±1.57	1.69±2.00	2.27±1.89	1.43±1.41	2.15±2.06	1.61±1.64	2.05±2.12	1.67±1.43	1.27±1.87	1.37±1.91
		13	0	29	43	25	50	38	43	43	29	33

Geometric mean with statistical deviation [GM ± SD] of the maximum fold-increase (d28/d-1 or d56/d-1) in antigen-specific IgG+ and IgA+ B_M_ cell responses for each cohort of vaccine candidates. The percentage of responders at each dose that showed a ≥2-fold higher B_M_ cell response over baseline is indicated in the second line of each cell. For sample sizes see legend under Fig 1.

At one or more of the three highest doses, 70% to 90% of the immunized subjects were responders with a ≥2-fold higher IgG+ B_M_ cell response to all 3 antigens while 100% of the WRSs3 vaccinees were responders with an IgG+ B_M_ cell response to IpaB at 10^4^ CFU (Table 1). The highest number (75%) of IgA+ B_M_ cell responders among WRSs2 vaccinees was seen with IVP as the antigen, seen here at 10^5^ CFU while the highest number of IgA+ B_M_ cell responders among WRSs3 vaccinees (57%) were specific to IVP and LPS, seen here at 10^4^ CFU (Table 1). About 10–30% of the placebos also showed a ≥2-fold increase in B_M_ cell responses.

### Kinetics of B_M_ cell responses after vaccination

**Fig 2:**
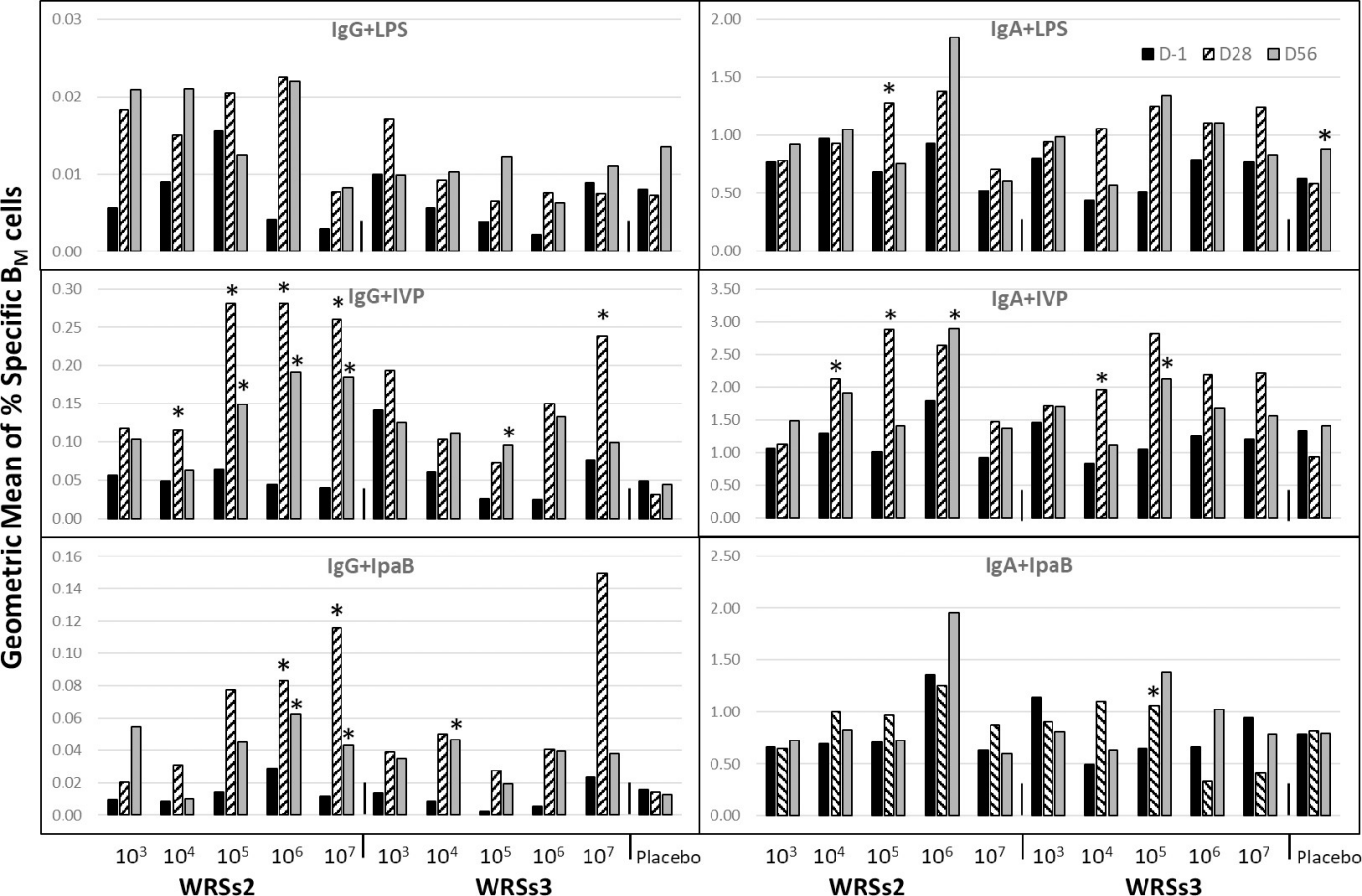
Kinetics of B_M_ cell responses after WRSs2 & WRSs3 vaccination. The geometric mean of the antigen-specific IgA+/IgG+ B_M_ cell response on days d-1, d28 and d56 is given on the Y-axis with the vaccine doses (CFU) on the X-axis. Antigen-specific IgA+/IgG+ B_M_ placebo responses are also included in each panel. A paired *t*-test comparing the magnitude of the B_M_ cell responses at d28 and/or d56 with d-1 was used to compute significance of the induced response (indicated by *asterisks).

When the geomean of the IgG+ and IgA+ B_M_ cell responses were compared at baseline and at post-vaccination, the geomean of the B_M_ cell responses to IVP and IpaB were highest at d28 (Fig 2). However, with LPS as the antigen, the B_M_ responses in some cases appeared higher at day 56 **(**Fig 2**)**. This feature becomes more evident when subject-specific kinetics of IgG+ and IgA+ B_M_ cell responses are observed at the three highest doses (S1A and S1B Fig). The IVP and IpaB-specific B_M_ cell responses were highest on day 28 and either remained elevated at day 56 or trended downwards, while with LPS, several subjects showed the highest response at day 56 post vaccination (S1A and S1B Fig). A paired *t*-test analysis indicated that the induced IVP-specific IgG+ B_M_ cell responses reached significance at 10^4^, 10^5^, 10^6^ and 10^7^ CFU among WRSs2 vaccinees and at 10^5^ and 10^7^ CFU among WRSs3 vaccinees (Fig 2, significance marked with asterisks*). Surprisingly, LPS-specific IgA+ B_M_ cell response among placebos also reached significance (*p* = 0.025) at d56.

### Correlations of B_M_ cell responses with responses to other immune parameters

At the three highest doses, 13 (57%), 12 (50%) and 8 (33%) subjects among WRSs2 vaccinees were IgA+ B_M_ cell responders and 12 (52%), 18 (78%) and 17 (74%) subjects were IgG+ B_M_ cell responders to LPS, IVP and IpaB respectively. The responder rates among WRSs3 vaccinees were 8 (36%), 9 (41%) and 9 (41%) subjects with IgA+ and 12 (55%), 17 (77%) and 18 (82%) subjects with IgG+ B_M_ cell response. Furthermore, at the same high doses, ≥75% of the vaccinees who responded positively to IVP and IpaB and 40–50% to LPS in serum/ALS/ASC assays, also showed an IgG+ B_M_ cell response to the same antigens. Similarly, >50% and 40% percent of the vaccinees who responded positively to LPS/IVP and IpaB respectively with an IgA+ response in serum/ASC/ALS/fecal assays also elicited an IgA+ B_M_ cell response to the same antigens.

Correlation determination between antigen-specific B_M_ cell responses and the systemic and mucosal immune responses previously described indicate that, in both groups of vaccinees, the best association is seen between IpaB-specific IgG+ B_M_ cell responses and IpaB-specific IgG+ responses in serum, ALS and ASC assays (Pearson’s coefficient or *r* = ≥ 0.45) with the correlation reaching significance (*p* ≤ 0.05) with IpaB-specific IgG+ ASCs in WRSs2 vaccinees and IpaB-specific IgG+ serum antibodies and ALS activity for both vaccine candidates.

## Discussion

A single, oral vaccination with several escalating doses of WRSs2 and WRsS3 demonstrated that while both candidates induced an array of antigen-specific systemic and mucosal immune responses that were dose dependent, the active responses appeared to be returning closer to the baseline within 4 weeks of vaccination [2, 3]. In this report we present data on B_M_ cell responses induced by the vaccine candidates up to d56 post-vaccination. The response varied greatly among vaccinated subjects suggesting that multiple factors may be responsible for this variability. Besides the limited sample sizes, another constraint of this study is the ELISpot assay itself that was carried out with frozen PBMCs which were not sorted for either B cells or B_M_ cells. Other contributory factors to the variability among vaccinees could be the role of gut microbiota and their metabolites at the time of oral vaccination [9]. Additionally, not much is known as to how B_M_ cells are generated after oral and subunit vaccinations, how existing pools of B_M_ cells are maintained and how B_M_ cell responses are affected after reinfection [4, 5]. While a single dose of WRSs2 and WRSs3 elicited IgG+/IgA+ B_M_ cell responses over baseline and over placebo responses in some vaccinees, a more extensive analysis in a larger group of subjects is needed to determine whether such a response, after one or more than one dose is required to predicate protection. An ongoing clinical trial, evaluating one and two doses of WRSs2 at 10^6^ CFU followed by a challenge with virulent *S*. *sonnei* strain 53G, is expected to provide additional information on the role of B_M_ cell responses during *Shigella* vaccination (NCT04242264).

Previous reports with live, attenuated *S*. *flexneri* 2a vaccine strains CVD 1204 and CVD 1208 demonstrated that LPS-specific IgG+ and IgA+ B_M_ cells were detected in circulation 28 days after oral vaccination similar to what has been described in this study with WRSs2 and WRSs3 [10, 11]. In the CVD 1204/1208 study the median percentages of antigen-specific SFC as a proportion of median total IgG+ SFCs increased from 0% at pre-vaccination to 0.02% and 0.03% post-vaccination for LPS and IpaB, respectively. B_M_ cell responses were seen exclusively among seroresponders and strong correlation were found between anti-LPS IgG+ B_M_ cell counts and peak serum anti-LPS IgG titers which did not change after adjusting for marker-specific cell populations [10, 11]. Such an association was not seen with WRSs2/WRSs3 vaccination.

That *Shigella* B_M_ cell responses can be a factor in protection was shown in an earlier study where frozen PBMCs from a set of “previously exposed” volunteers and naïve controls who were challenged with a virulent strain were evaluated for B_M_ cell responses [12]. The “previously exposed” group consisted of volunteers administered multiple oral doses of a live vaccine candidate, EcSf2a-2, followed by a challenge with *S*. *flexneri* 2a strain 2457T [13]. The vaccine conferred a modest ~30% efficacy. A subset of these previously exposed volunteers who developed gastrointestinal symptoms after challenge participated in a second re-challenge study with 2457T along with a second group of naïve volunteers [14]. In this case the protective efficacy of prior exposure to 2457T reached 70% [14]. Available cryopreserved PBMCs from the pre-exposed group and the rechallenged subjects were used to perform B_M_ assays to determine if any correlations could be made between B_M_ cells and the observed improved efficacy [12]. Post-challenge LPS-specific IgA+ B_M_ cell responses negatively correlated with disease severity in pre-exposed and re-challenged vaccinated groups but not in the naïve-challenged group [12]. In contrast to LPS, pre-challenge but not post-challenge IpaB-specific IgA+ B_M_ cells among pre-exposed volunteers negatively correlated with disease indices. A trend towards a negative correlation of pre- and post-challenge IgG+ IpaB-specific B_M_ cell response with disease severity was also observed. Although we have no efficacy data, it is interesting to note that the best correlation after WRSs2/WRSs3 vaccination was observed between IgG+ IpaB-specific B_M_ cell responses and IgG+ responses to IpaB in serum, ALS and ASC assays. In an immunoprofiling study with sera from the EcSf2a-2 efficacy trial, pre-challenge IpaB-specific IgG+ and IgA+ levels and Fcγ receptor binding negatively correlated with all measured shigellosis symptoms following challenge [15].

It is generally accepted that an initial infection with *Shigella* causing disease will be protective against a subsequent reinfection with the same serotype. In a controlled human infection model (CHIM) study with 53G, a virulent *S*. *sonnei* strain, IgG+ and IgA+ B_M_ cell responses to LPS and IVP were elevated over baseline at day 28 and remained so until day 56 [16]. With the exception of LPS-specific IgG+ B_M_ cell responses, volunteers with shigellosis (presumptively protective state) had larger rises in LPS and IVP-specific IgA+ and IVP-specific IgG+ B_M_ ALS titers than volunteers without disease [16]. Furthermore, increased baseline titers of LPS-specific serum IgA together with LPS-specific IgA+ B_M_ cell responses appeared to coincide with volunteers not progressing to shigellosis. These results suggested that LPS-specific serum IgA, and not serum IgG as is often considered, as well as baseline levels of IgA-secreting B_M_ cells may be alternate predictors of resistance to shigellosis [16].

Immunoprofiling using a parenterally administered bioconjugate *S*. *flexneri* 2a subunit vaccine candidate, Flexyn2, was compared with immune responses from the CHIM study with orally administered 53G [17, 18]. Flexyn2 induced significant increase in LPS-specific IgG+ B_M_ cell responses by day 56 which further increased after challenge with a virulent *S*. *flexneri* 2a strain 2457T [17, 18]. IgG+ B_M_ responses in vaccinated subjects were comparable to the IgG+ B_M_ cell responses in placebo recipients post-challenge. IgG+ and IgA+ B_M_ cell responses correlated best with each other; however, IgG+ B_M_ cell responses also correlated with serum IgG/IgA responses. IgA+ B_M_ cell responses correlated best with serum IgA responses as well as with α4β7- IgA responses [17, 18]. Placebo subjects not progressing to shigellosis were found to have significantly higher LPS-specific IgA+ B_M_ cell responses on the day of challenge as compared to placebos who developed shigellosis, confirming the protective role of LPS-specific IgA+ B_M_ cell responses that was also reported in the 53G CHIM study described above. Conclusions derived from these studies indicate that parenterally immunized subjects protected after challenge show a robust LPS-specific systemic/memory immune responses including higher IgG+ B_M_ cell responses as compared to orally challenged subjects developing shigellosis who showed a stronger correlation with LPS-specific mucosal responses as well as higher IgA+ B_M_ cell responses [18].

Thus, B_M_ cell generation as well as the magnitude of the B_M_ responses that is critical for effective vaccination and protection, has made B_M_ cell measurements a consensus second-tier immune assay to be performed during future *Shigella* vaccination and challenge studies [19, 20]. Since *Shigella* vaccination is critical for children in poorly resourced countries, it is also important to perform immunoprofiling studies in infants, children and adults in highly endemic regions to determine pre-existing immunological parameters against LPS and protein antigens, including B_M_ cell responses, that could serve as a correlate of a reduced risk for disease or protection under natural conditions of exposure [21, 22].

## Supporting information

S1 Fig **A.** Subject-specific kinetics of the IgG+ B_M_ cell responses at the 3 highest doses. Antigen-specific IgG+ B_M_ cells for each subject vaccinated at 10^5^, 10^6^ and 10^7^ CFU doses with WRSs2 and WRSs3 are shown on the Y-axis against baseline and post vaccination days (d-1, d28 and d56). The line shading from the lightest (10^5^ CFU) to intermediate (10^6^ CFU) to the darkest (10^7^ CFU) denotes the three highest doses of vaccination. Placebo responses are shown in the right hand panels for each antigen. **B.** Subject-specific kinetics of the IgA+ B_M_ cell responses at the 3 highest doses. The percentage of antigen-specific IgA+ B_M_ cells for each subject vaccinated at 10^5^, 10^6^ and 10^7^ CFU doses with WRSs2 and WRSs3 are shown on the Y-axis against pre- and post-vaccination days as shown in S1A Fig. The line shading from the lightest (10^5^ CFU) to intermediate (10^6^ CFU) to the darkest (10^7^ CFU) denotes the doses in CFU. Placebo responses are shown in the right hand panels for each antigen.
(ZIP)
