# Supplementary figures and images for: B memory cell responses to LPS, IVP and IpaB antigen after oral vaccination with Shigella sonnei vaccine candidates WRSs2 and WRSs3
Source: PLoS One. 2024 Jan 17;19(1):e0290987. doi: 10.1371/journal.pone.0290987 (PMC10793881; doi:10.1371/journal.pone.0290987)

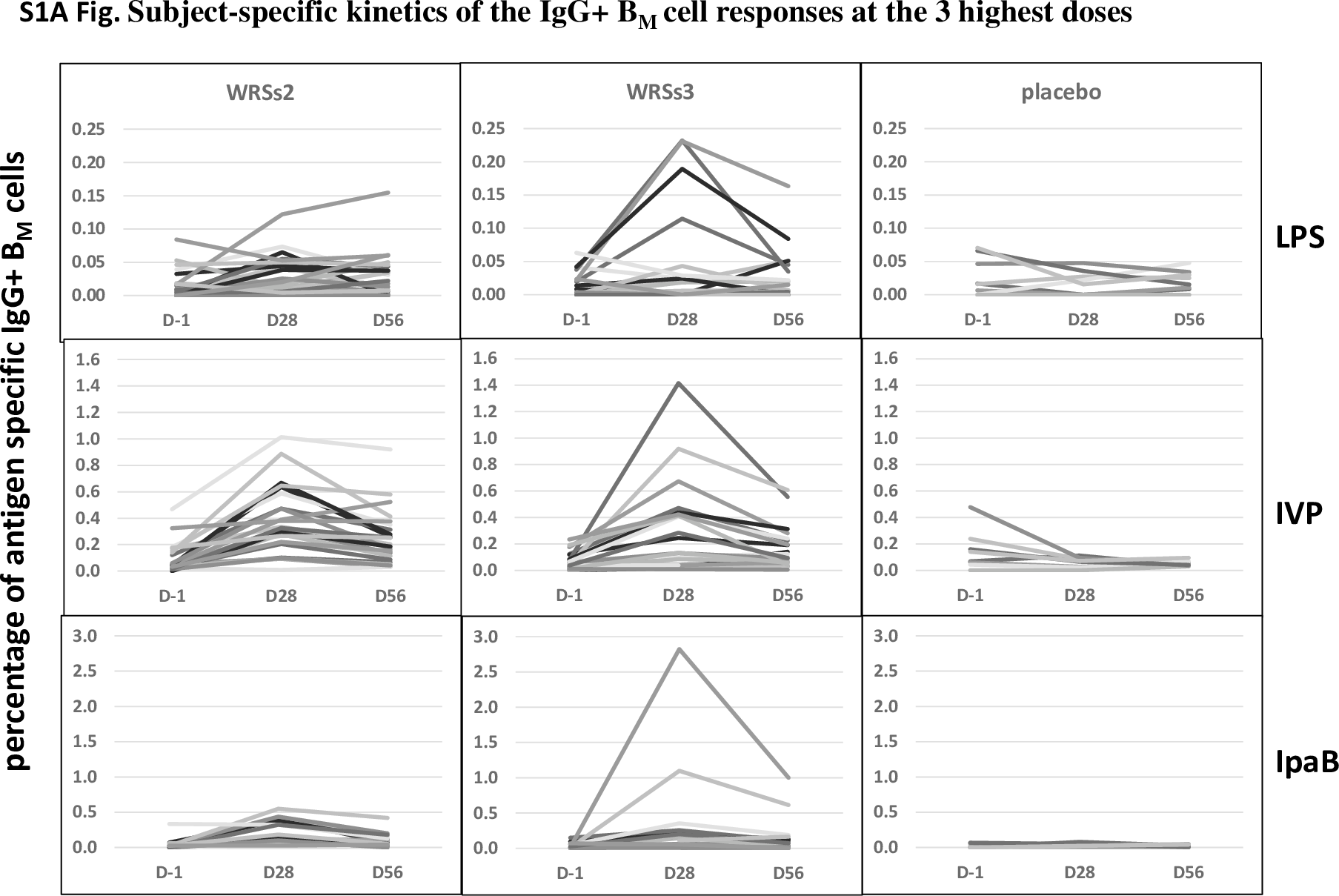

Supplement: S1 Fig — A. Subject-specific kinetics of the IgG+ BM cell responses at the 3 highest doses. Antigen-specific IgG+ BM cells for each subject vaccinated at 105, 106 and 107 CFU doses with WRSs2 and WRSs3 are shown on the Y-axis against baseline and post vaccination days (d-1, d28 and d56). The line shading from the lightest (105 CFU) to intermediate (106 CFU) to the darkest (107 CFU) denotes the three highest doses of vaccination. Placebo responses are shown in the right hand panels for each antigen. B. Subject-specific kinetics of the IgA+ BM cell responses at the 3 highest doses. The percentage of antigen-specific IgA+ BM cells for each subject vaccinated at 105, 106 and 107 CFU doses with WRSs2 and WRSs3 are shown on the Y-axis against pre- and post-vaccination days as shown in S1A Fig. The line shading from the lightest (105 CFU) to intermediate (106 CFU) to the darkest (107 CFU) denotes the doses in CFU. Placebo responses are shown in the right hand panels for each antigen. (ZIP) [file pone.0290987.s001.zip › S1A Fig-3-29.tif]

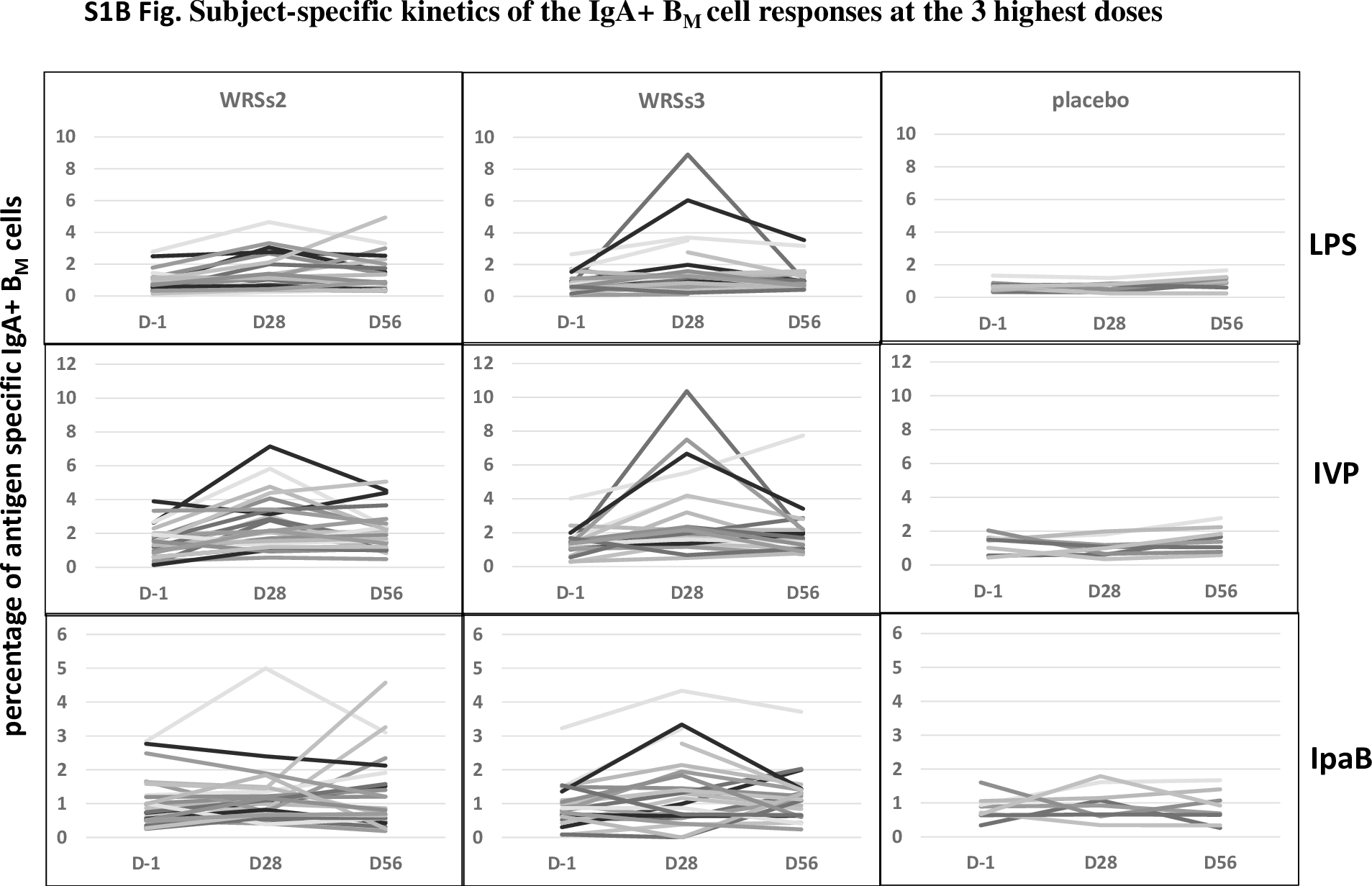

Supplement: S1 Fig — A. Subject-specific kinetics of the IgG+ BM cell responses at the 3 highest doses. Antigen-specific IgG+ BM cells for each subject vaccinated at 105, 106 and 107 CFU doses with WRSs2 and WRSs3 are shown on the Y-axis against baseline and post vaccination days (d-1, d28 and d56). The line shading from the lightest (105 CFU) to intermediate (106 CFU) to the darkest (107 CFU) denotes the three highest doses of vaccination. Placebo responses are shown in the right hand panels for each antigen. B. Subject-specific kinetics of the IgA+ BM cell responses at the 3 highest doses. The percentage of antigen-specific IgA+ BM cells for each subject vaccinated at 105, 106 and 107 CFU doses with WRSs2 and WRSs3 are shown on the Y-axis against pre- and post-vaccination days as shown in S1A Fig. The line shading from the lightest (105 CFU) to intermediate (106 CFU) to the darkest (107 CFU) denotes the doses in CFU. Placebo responses are shown in the right hand panels for each antigen. (ZIP) [file pone.0290987.s001.zip › S1B Fig.-3-29.tif]
